# Identical synchronization of a non-autonomous unified chaotic system with continuous periodic switch

**DOI:** 10.1186/s40064-016-3299-6

**Published:** 2016-09-27

**Authors:** Behnaz Koocheck Shooshtari, AbdolMohammad Forouzanfar, MohammadReza Molaei

**Affiliations:** 1 Department of Mathematics, Shahid Chamran Univesity of Ahvaz, Ahvaz, Iran; 2 Department of Mathematics, Shahid Bahonar University of Kerman, Kerman, Iran; 3 Center of Excellence on Modeling and Control Systems, Ferdowsi University, Mashhad, Iran

**Keywords:** Unified chaotic system, Chen system, Lorenz system, Synchronization, Stability, Instantaneous equilibrium point, Primary 34D06, 34D20, 34D08, Secondary 37L30

## Abstract

In this article a non-autonomous unified chaotic system with continuous periodic switch between the Chen and Lorenz systems is introduced. Dynamical behaviors of this system are investigated. We consider the identical (complete) synchronization of the bi-directionally coupled between two identical systems of this type and then analyze its stability by estimating the entire Lyapunov characteristic exponent spectrum. Numerical and graphical works are done with Mathematica.

## Background

Many real phenomena can be described by deterministic ordinary nonlinear differential equations (Hilborn 2000; Parker and Chua 1989; Wiggins 1990). Explicit expression of solutions are unavailable for the most interesting systems, thus we can use of numerical procedures for approximating solutions. This process may be applied even for systems with a wide range of chaotic behaviors. Chaotic systems are well-known for their complex nonlinear behaviors and they possess certain characteristics as sensitivity to initial conditions. These systems have been studied in various fields such as physics, chemistry, engineering, biology and information sciences (Hilborn 2000; Brin and Stuck 2003; Strogatz 1994). Recently, the Lie algebra method has used to obtain exact solutions for some nonlinear ordinary differential equations (Shang 2012, 2013, 2015b).

Since 1990, researchers have realized that chaotic systems can be synchronized. There are very noticeable results reported about chaos synchronization in secure communication, image processing and other fields. Synchronization of chaos is a phenomenon that may happen when some dissipative chaotic systems are coupled. It seems that Chaotic systems oppose with synchronization, because two identical chaotic systems with nearly the same initial conditions have trajectories in phase space which diverge quickly. By synchronization, the trajectories of one of the systems will converge to the same values as the other (Boccaletti et al. 2002; Brown and Kocarev 2000; Gonzalez Miranda 2004; Molaei 2011; Singh and Handa 2012; Wikipedia). In fact, the synchronization appears to be structurally stable (Pecora and Carroll 1990). By depending on the nature of the interacted systems and of their coupling configuration, the forms of synchronization may be different. Some of them are identical synchronization, generalized synchronization, phase synchronization, anticipated and lag synchronization, and other kinds of synchronization schema as generalized lag synchronization (Huanga et al. 2009) and adaptive pinning control for the projective synchronization (Xiao et al. 2012) that are the recent development for the synchronization of chaos. Many different forms of synchronization are possible in unidirectional or bidirectional coupling configuration (Brown and Kocarev 2000; Gonzalez Miranda 2004; Pikovsky et al. 2001; Singh and Handa 2012; Wikipedia). In identical (complete) synchronization, two dynamical systems have the same behavior at the same time that restricted to a hyperplane, the synchronization manifold, in the phase space (Carroll et al. 1997; Tarai et al. 2009). Therefore in studying the synchronization, there are two fundamental investigations: finding the synchronization manifold and determining its stability (Carroll et al. 1997; Fujisaka and Yamada 1983a, b).

We mention that there is different between synchronization and consensus. In the consensus problem (Shang 2015a), we change a system with parameters to a system with simple parameters so that these two systems have the same behavior but in the synchronization problem we do not need to change the parameters to simple parameters. Consensus systems usually require linear and identical dynamics for uncoupled systems. Consensus problems have many applications in engineering, social and biological fields.

There is an additional complication to synchronization of chaotic systems when they are non-autonomous (They have some explicit time dependence.). One of the reason for considering non-autonomous systems is that in the some of physical systems, the parameters associated with these systems may vary with time.

In this article we introduce a non-autonomous unified chaotic system with continuous periodic switch between the Chen and Lorenz systems. This system exhibits abundant wonderful dynamics for different values of its parameter that in very beautiful figures will be shown. Many studies in the future can be discerned about this system and its applications. For recognizing better this system, we will consider the general properties of its dynamical behaviors as symmetry, dissipativity, existence of attractor and instantaneous equilibria with their stability. Then we will discuss the identical synchronization between two systems of this type with bidirectional coupling configurations; started at slightly different initial conditions. Finally we will study the synchronized motion and its stability by estimating the Lyapunov characteristic exponents (LCE) spectrum which are shown in various figures of two and three dimentional.

The article is organized as follows: In the next section we will consider unified chaotic system. Then in section three we will introduce a non-autonomous unified chaotic system. Its dynamical behaviors will be studied in section four. Lyapunov characteristic exponents (LCE) of it, will be considered in sections five and six. In section seven, we will study the identical synchronization and its stability with Mathematica (Gray 1998) implementation for calculating LCE spectrum. Finally, section eight will be the conclusion.

## Unified chaotic system

In many natural phenomena, an nth-order autonomous continuous-time dynamical system is defined by differential equation

1 $$\begin{aligned} \dot{X}=F(X),\quad X(t_0 )=X_0, \end{aligned}$$

where $\dot{X}=\frac{dX}{dt}, X(t)\in \mathbb {R}^n$ is the state vector at time t, and $F:\mathbb {R}^n \rightarrow \mathbb {R}^n$ is a $C^1$ (the space of continuously differentiable functions) function. The solution of system is often written as $f^t (X )$.

The one-parameter family of mapping $f^t: \mathbb {R}^n\rightarrow \mathbb {R}^n$, satisfies the two conditions, $f^{t_1+t_2 }=f^{t_1}o f^{t_2 }$ and $f^0 (X)=X$ is called the flow. The set of points $\{ f^t (X_0 ): t\in \mathbb {R}\}$ is called the trajectory through $X_0$.

An nth-order non-autonomous continuous-time dynamical system is defined by differential equation

2 $$\begin{aligned} \dot{X}=F(X,t),\quad X{(t_0 )}=X_0. \end{aligned}$$

In this case the vector field depends on time and the solution of system passing through $X_0$ at time $t_{0}$ is denoted by $f^t (X_0,t_0 )$ (Parker and Chua 1989).

Since Lorenz found the first chaotic attractor (Lorenz 1963), chaos has been extensively studied in science, engineering, physics and mathematics (Pecora and Carroll 1990; Carroll et al. 1997). In 1999, Chen found another similar but topologically not equivalent chaotic attractor (Chen and Ueta 1999). In 2002, Lu et al. produced a unified chaotic system that not only bridges the gap between the Lorenz and the Chen system but also represent the entire family of chaotic systems between them (Lu et al. 2002). The unified chaotic system, for $\alpha \in [0,1]$ is described by

3 $$\begin{aligned} {\left\{ \begin{array}{ll} \dot{x}=(25\alpha +10)(y-x); \\ \dot{y}=(28-35\alpha )x-xz+(29\alpha -1)y; \\ \dot{z}=xy-\frac{(\alpha +8)}{3} z. \end{array}\right. } \end{aligned}$$

In 2004, Junan and Xiaoqun introduced a non-autonomous unified chaotic system with continuous periodic switch between the Lorenz and Chen systems under inspiration of the unified chaotic system (3) with $\alpha =sin^2 (\omega t)$ and $\omega$ is an adjustable parameter system that was called switching system (Junan and Xiaoqun 2004).

Currently it is being actively discussed the question of the equivalence of various Lorenz-like systems and the possibility of universal consideration of their behavior in view of the possibility of reduction of such systems to the same form with the help of various transformations. Leonov and Kuznetsov have discussed the differences and similarities in the analysis of these systems and they have shown that the Chen and the Lu systems stimulate for the development of new methods for the analysis of chaotic systems (Leonov and Kuznetsov 2015).

## The another unified chaotic system with continuous periodic switch

**Fig. 1:**
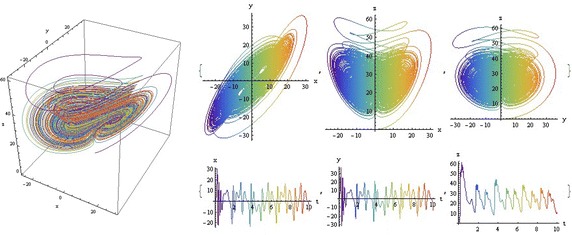
$$\omega =0$$

**Fig. 2:**
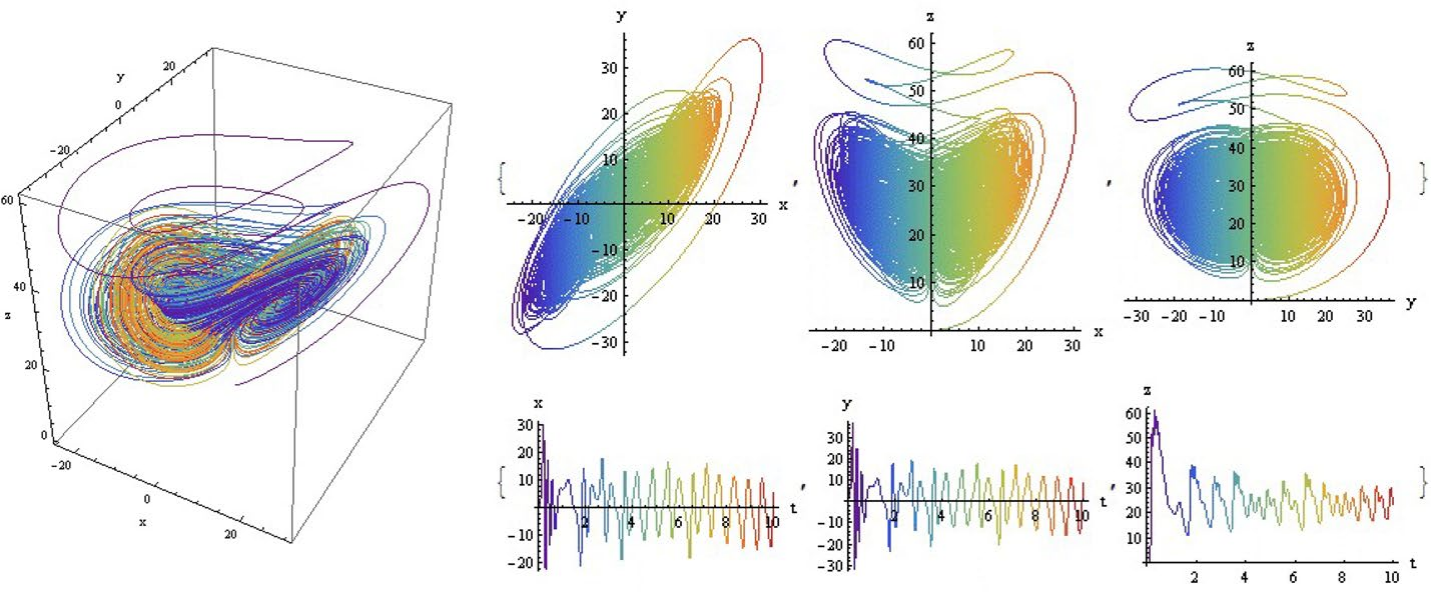
$$\omega =0.01$$

**Fig. 3:**
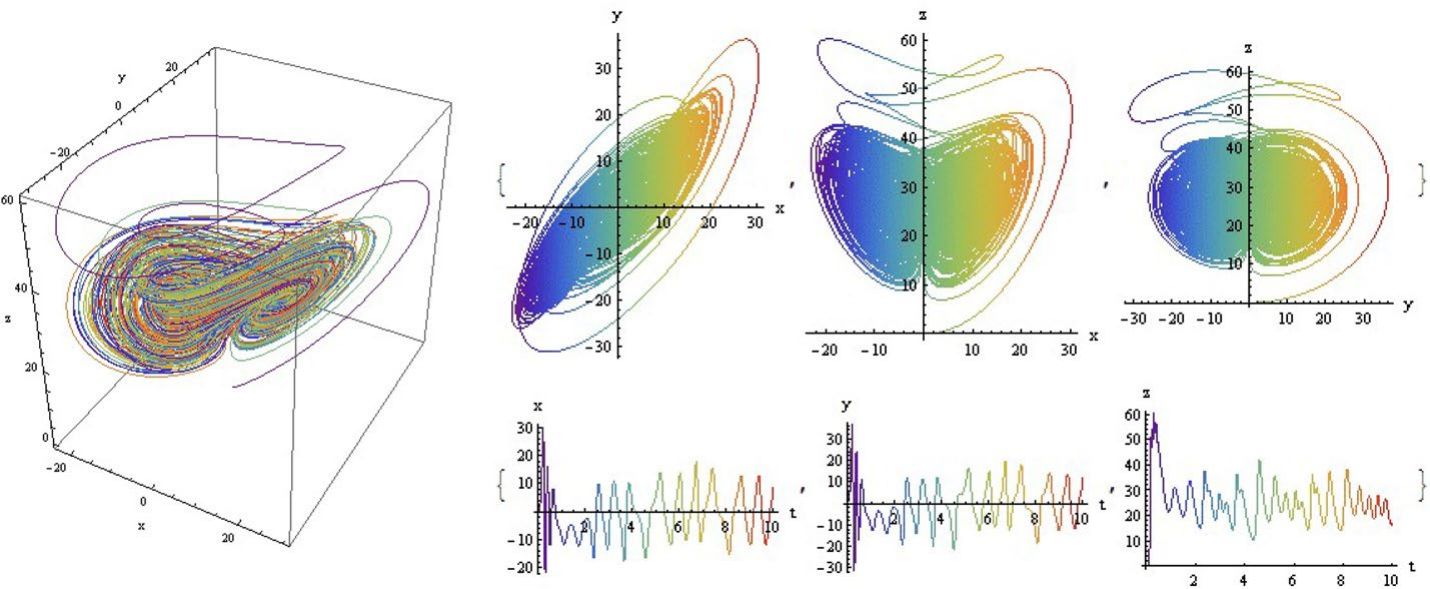
$$\omega =1$$

**Fig. 4:**
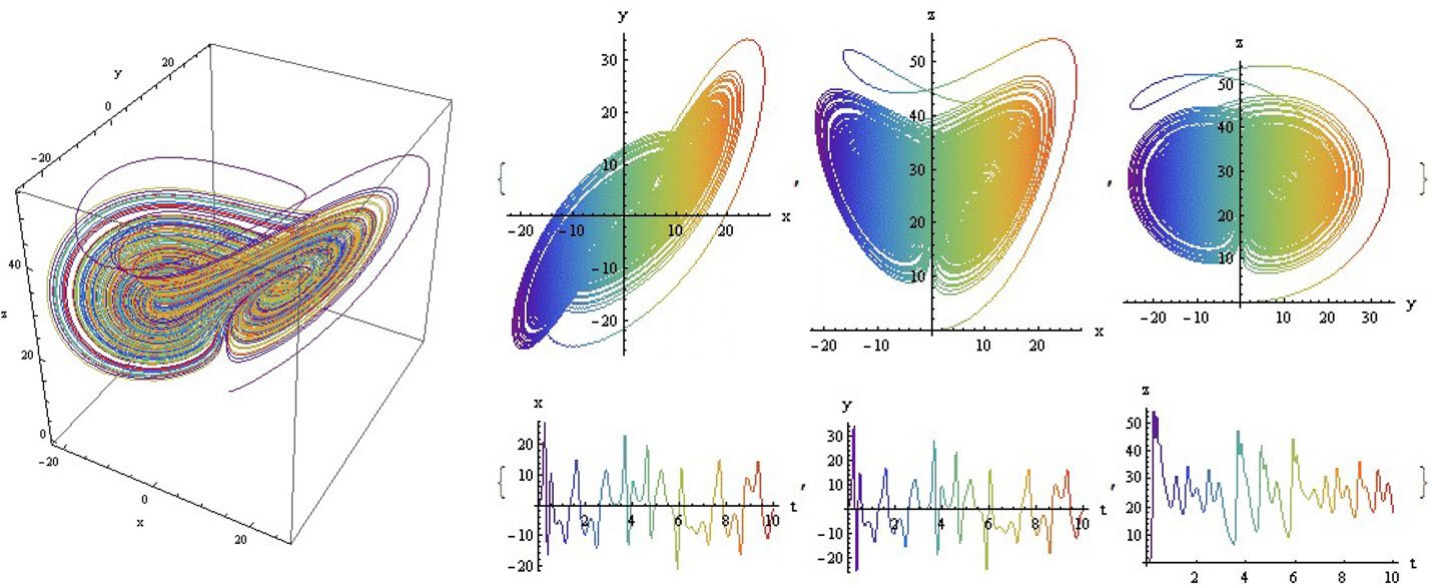
$$\omega =1000$$

We introduce a non-autonomous unified chaotic system with continuous periodic switch between Chen and Lorenz systems under inspiration of the unified chaotic system (3), which is described as follows:

4 $$\begin{aligned} {\left\{ \begin{array}{ll} \dot{x}=(25 cos^2(\omega t )+10)(y-x); \\ \dot{y}=(28-35cos^2(\omega t))x - xz +(29 cos^2(\omega t)-1)y;\\ \dot{z}=xy-\frac{ cos^2(\omega t )+8}{3} z. \end{array}\right. } \end{aligned}$$

where $\omega$ is adjustable parameter of system (4). When the system evolves, t increases, the system (4) switches continuously between the Chen and Lorenz systems. The frequency of switching is controlled by the parameter $\omega$. This system exhibits abundant wonderful dynamics that for $\omega =0,0.01,1,1000$ are shown in Figs. 1, 2, 3 and 4.

In these figures we demonstrate the three dimentional chaotic attractor of system, projections of the chaotic attractor to three orthogonal planes and the time series obtained from the time evolution of the variables *x*(*t*), *y*(*t*) and *z*(*t*).

For $\omega = 0$, we will obtain the Chen system from system (4), while, for this value of $\omega$, one can obtain the Lorenz system from switching system. It might be suggested that the system (4) is the dual to the switching system in Junan and Xiaoqun (2004).

## Dynamical behaviors

### Symmetry

The system (4) has a natural symmetry under the coordinates transform,

$$\begin{aligned} (x,y,z) \rightarrow (-x,-y,z), \end{aligned}$$

for all values $\omega$. Furthermore, the trajectory on the z-axis tends to the origin as $t\rightarrow \infty$, since for such a trajectory, we have:

$$\begin{aligned} \frac{dx}{dt}=\frac{dy}{dt}=0,\quad \frac{dz}{dt}=-\frac{cos^2 (\omega t)+8}{3} \end{aligned}$$

###  Dissipative property and the existence of attractor

The system (4) is dissipative: volumes in phase space contract under the flow. For understanding that how to do volumes evolve, in general, we consider a three-dimensional system $\dot{X}=F(X)$ (Ott 1994; Strogatz 1994). Choose an arbitrary closed surface *S*(*t*) of volume *V*(*t*) in phase space. Suppose the points on *S* be as initial conditions for trajectories, and they evolve for an infinitesimal time *dt*. Then the surface *S* evolves in to a new surface $S(t+dt)$ of volume $V(t+dt)$. If *n* denotes the outward normal on *S*, then *F*.*n* is the outward normal component of velocity (Because *F* is the instantaneous velocity of the points). Therefore in time *dt*, a patch of erea *dA* sweeps out a volume (*F*.*ndt*)*dA* and we obtain,

$$\begin{aligned} V(t+dt)=V(t)+ \int _S(F.n~ dt)~dA, \end{aligned}$$

and so,

$$\begin{aligned} \dot{V}=\frac{V(t+dt)-V(t)}{dt}=\int _S~ F. n ~dA, \end{aligned}$$

then by the divergence theorem, we have,

$$\begin{aligned} \dot{V}=\int _V \nabla . F dV. \end{aligned}$$

For our system,

$$\begin{aligned} \nabla \cdot F=\frac{\partial \dot{x}}{\partial x}+\frac{\partial \dot{y}}{\partial y}+\frac{\partial \dot{z}}{\partial z}=-(25 cos^2 (\omega t)+10)+(29 cos^2 (\omega t)-1)-\frac{cos^2 (\omega t)+8}{3}, \end{aligned}$$

therefore,

$$\begin{aligned} \nabla . F= - \frac{41-11cos^2 (\omega t)}{3}< 0, \quad t\ge 0. \end{aligned}$$

Since the divergence is constant with respect to the state vector, we have,

$$\begin{aligned} \dot{V}=-\frac{41-11cos^2 (\omega t)}{3} V, \end{aligned}$$

which has solution,

$$\begin{aligned} V(t)=V(0) \exp \left( -\frac{41-11cos^2 (\omega t)}{3}\right) . \end{aligned}$$

Thus volumes in the phase space shrink to zero with an exponential rate independent of *x*, *y*, *z* as $t\rightarrow \infty$ and the system (4) is dissipative for all $\omega$. This does not imply that each small volume shrinks to a point but may imply become flattened into a surface. Therefore all trajectories ultimately become confined to a specific subspace with zero volume, and the motion of system asymptotically settles onto an attractor.

### Instantaneous equilibria and stability

Suppose $t_0$ is arbitrary and constant, for every time $t \ge t_0$, from system (4) we can solve the bellow system:

5 $$\begin{aligned} {\left\{ \begin{array}{ll} \left( 25 cos^2(\omega t) +10\right) (y-x)= 0; \\ \left( 28-35cos^2(\omega t)\right) x -xz +(29 cos^2(\omega t)-1)y= 0; \\ xy-\frac{ cos^2(\omega t) +8}{3} z = 0. \end{array}\right. } \end{aligned}$$

It can be verified that system (5) has three solutions: $S_0 (0,0,0)$,

$$\begin{aligned}&S_+ \left( \sqrt{72-7 cos^2(\omega t )-2 cos^4(\omega t)}, \sqrt{72-7 cos^2(\omega t )-2 cos^4(\omega t)},-3(-9+2 cos^2(\omega t))\right) ,\\&S_ - \left( -\sqrt{72-7 cos^2(\omega t )-2 cos^4(\omega t)},- \sqrt{72-7 cos^2(\omega t )-2 cos^4(\omega t)},-3(-9+2 cos^2(\omega t))\right) , \end{aligned}$$

which we call them instantaneous equilibrium points. We see that $S_+$ and $S_-$, are symmetrically placed with respect to the z-axis. Jacobian matrix in linearizing the system (4) is:

$$\begin{aligned} J = \left( \begin{matrix} -10-25 cos^2(\omega t ) &{}10+25 cos^2(\omega t ) &{} 0 \\ 28-z-35 cos^2(\omega t ) &{} -1+29 cos^2(\omega t )&{} -x\\ y&{}x&{}\frac{1}{3}(-8- cos^2(\omega t)) \end{matrix}\right) . \end{aligned}$$

The characteristic equation of matrix *J* at $S_ 0$ is,

$$\begin{aligned} f(\lambda )=(\lambda ^2+(11-4 cos^2(\omega t )) \lambda +(25 cos^2(\omega t)+10) (6 cos^2(\omega t )-27)\left( \lambda +\frac{ cos^2(\omega t )+8}{3}\right) . \end{aligned}$$

Thus from $f(\lambda )= 0$, one of eigenvalues is $\lambda _1=-\frac{cos^2(\omega t )+8}{3}< 0$ and the others satisfy $\lambda _2>0>\lambda _3$. So, $S_ 0$ is a saddle point in the phase space.

The characteristic equation of matrix J at the other instantaneous equilibria is:

$$\begin{aligned} f( \lambda )&= \lambda ^3+A \lambda ^2+B \lambda +C= 0,\quad{\text { with}}~~ A=\frac{41-11cos^2(\omega t)}{3},\\ B& = \frac{(38-10 cos^2(\omega t ))( cos^2(\omega t )+8)}{3}, \end{aligned}$$

and

$$C=2(25 cos^2(\omega t )+10)( cos^2(\omega t )+8)(-2 cos^2(\omega t )+9).$$

By the Routh-Hurwitz stability criterion, a necessary and sufficient condition that each root of $f(\lambda )$ have negative real part is that

$$\begin{aligned} A> 0, AB-C> 0, \quad (AB-C)C> 0. \end{aligned}$$

**Fig. 5:**
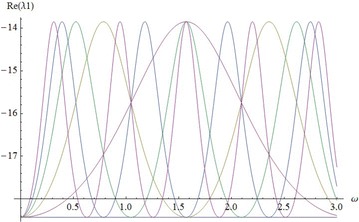
Real $$\lambda _1$$, $$t=0,\ldots ,5$$

**Fig. 6:**
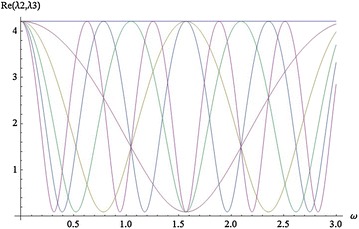
Real $$\lambda _2$$, $$\lambda _3$$, $$t=0,\ldots ,5$$

These conditions are satisfied if and only if $- 0.4< cos^2(\omega t )<- 0.0 13681$. But this is impossible. Therefore the three instantaneous equilibria of system (4) are unstable for all values of parameter. The correlation between real parts of eigenvalues and parameter $\omega$ for $t= 0, \ldots ,5$ are shown in Figs. 5 and 6.

##  Lyapunov characteristic exponent

Lyapunov characteristic exponents (LCE) give the rate of exponential divergence from perturbed initial conditions in a phase space. We consider an infinitesimal hypersphere of initial conditions in the phase space. The effect of the dynamics for sufficiently short time scales, will turn this hypersphere to the shape of a hyperellipsoid, contracted along one direction and stretched along another. This is because the rate of divergence of the trajectories that start in the points initially in hypersphere will be different along different directions. The asymptotically rate of expansion of the largest axis, is measured by the largest LCE that is corresponded to the most unstable direction. Since a positive LCE indicates expansion, the existence of it distinguishes strange attractors from non-chaotic attractors. On the other hand for any attractor other than a fixed point, one LCE must be zero and the sum of the LCEs of an attractor of a dissipative system must be negative (Parker and Chua 1989). Therefore a strange attractor must have at least three LCEs that their numbers are equal to the dimension of the phase space. For a chaotic system, the spectrum of its LCEs in decreasing order by magnitude is $\lambda _1\ge \lambda _2\ge \cdots \ge \lambda _n$. Since the direction of the axes of the ellipsoid change with time, therefore there is no well-defined direction associated to each LCE.

Suppose $X_ 0$ and $X_ 0 +\delta X_ 0$ are two nearby points in the phase space where $\delta X_ 0$ is a small perturbation of the initial state. The perturbation $\delta X_t$, after a time *t*, will become:

6 $$\begin{aligned} \delta X_t= f^t (X_ 0 +\delta X_ 0)-f^t (X_ 0)=D_{X_ 0 } f^t{(X_ 0)} \cdot \delta X_ 0, \end{aligned}$$

where $f^t (X_ 0)$ and $f^t (X_0+\delta X_ 0)$ are images of two points under the flow and the last term is obtained by linearizing $f^t$. Now, the average exponential rate of divergence of the two trajectories is defined by:

7 $$\begin{aligned} \lambda (X_0,\delta X_ 0 )=\lim _{t\rightarrow \infty }\frac{1}{t} ln\frac{||\delta X_t||}{ || \delta X_ 0 || }=\lim _{t\rightarrow \infty } \frac{1}{t} ln || D_{X_ 0} f^t (X_ 0)\cdot \delta X_ 0 ||. \end{aligned}$$

This limit exists, for almost all points, and for almost all tangent vectors in basin of attraction and is called the largest LCE, $\lambda _1$ (Sandri 1996).

Definition (7) refers to LCEs of order one of vectors. In general (Parker and Chua 1989; Sandri 1996), the LCEs of order $1\le p\le n$, is defined by

8 $$\begin{aligned} \lambda ^p (X_ 0, M_ 0)=\lim _{t\rightarrow \infty }\frac{1}{t} ln \left[ V ol^p (D_{X_ 0} f^t (M_ 0 ))\right] , \end{aligned}$$

where $Vol^p$ is the *p*-dimensional volume of a parallelepiped $M_ 0$ in the tangent space whose edges are the *p* vectors $u_1,u_2,\ldots ,u_p$. These LCEs describe the average rate of change of a $Vol ^p$. There exist *p* linearly independent vectors $u_1,u_2,\ldots ,u_p$ such that

9 $$\begin{aligned} \lambda ^p (X_ 0 ,M_ 0 )=\lambda _1+\lambda _2+\cdots +\lambda _p, \end{aligned}$$

If $\{w_1,w_2,\ldots ,w_p\}$ be the set of orthogonal vectors of $\{u_1,u_2,\ldots ,u_p\}$, obtained by Gramm-Schmidt method, then volume of the parallelepiped spanned by $\{u_1,u_2,\ldots ,u_p\}$ is

$$\begin{aligned} Vol \{u_1,u_2,\ldots ,u_p \}= || w_1 ||\ldots ||w_p ||. \end{aligned}$$

##  Estimation of the entire LCE spectrum

Consider the n-th order system

10 $$\begin{aligned} \dot{X}=F(X,t) , \end{aligned}$$

with $X_0$ , as an initial condition in the basin of attractor. According Parker and Chua (1989), one can verify that the vector defined in (6), satisfies in the variational equation:

11 $$\begin{aligned} \dot{\Phi _t} (X_ 0 )=D_X F\left( f^t (X_ 0 )\right) \cdot \Phi _t (X_ 0 ),\quad \Phi _ 0 (X_ 0 )=I, \end{aligned}$$

where $\Phi _t (X_ 0)$ is the derivative with respect to $X_ 0$ of $f^t$ at $X_ 0$, that is, $\Phi _t (X_ 0)= D_{X_ 0 } f^t (X_ 0 )$. Equation (11) is a matrix-valued time-varying linear differential equation whose coefficients depend on the evolution of the original system (10). It is the linearization of the vector field along the trajectory $f^t (X_ 0 ,t_ 0 )$. Initial condition is the identity matrix *I*. Since the variational equation (11) depends on both $f^t$ and $\Phi _t$, they must be calculated at the same time. To perform this work, we append the variational equation to the original system for obtaining the new combined system:

12 $$\begin{aligned} \left( \begin{array}{cc} \dot{X}\\ \dot{ \Phi }\\ \end{array} \right) =\left( \begin{array}{cc} F(X)\\ D_X F{(X)}.\Phi \\ \end{array} \right) ,\quad \left( \begin{array}{cc} X{(t_ 0 ) }\\ \Phi {( t_ 0 )}\\ \end{array} \right) =\left( \begin{array}{cc} X_0\\ I\\ \end{array} \right) \end{aligned}$$

For non-autonomous system $\dot{X}=F(X,t)$, it will be sufficient to treat *t* as an additional dependent variable with the trivial evolution equation $\dot{t}=1$, and we will rewrite every non-autonomous system as an autonomous system (Parker and Chua 1989; Sandri 1996)

13 $$\begin{aligned} \dot{X}=F(X,t),\quad \dot{t} =1. \end{aligned}$$

For the calculation of LCEs, via the algorithm of Benettin et al. (1980a, b), Sandri (1996), we choose an initial vector $X_ 0$, an $n\times n$ matrix $M_ 0 =[u_1^{( 0 )},\ldots ,u_n^{( 0 )} ]$, and by Gramm-Schmidt method, we obtain the matrix $N_ 0 =[v_1^{( 0 )},\ldots ,v_n^{( 0 )} ]$ of orthonormal vectors corresponding to $M_ 0$. Now integrate the variational equation (12) by using $$\left( \begin{array}{cc} X_ 0 \\ N_ 0\\ \end{array} \right)$$ in a short interval *T* of time, for obtaining $X_1=f^t (X_ 0 )$ and

$$\begin{aligned} M_1=\left[ u_1^{(1)},\ldots ,u_n^{(1)} \right] =D_{X_ 0 } f^t (M_ 0 )=\Phi _T (X_ 0 )\cdot \left[ u_1^{( 0 )},\ldots ,u_n^{( 0 )} \right] . \end{aligned}$$

Again, we obtain the matrix $N_1=[v_1^{(1)},\ldots ,v_n^{(1)}]$ of orthonormal vectors correspond to $M_1$ and integrate the Eq. (12) by using $$\left( \begin{array}{cc} X_ 1 \\ N_ 1\\ \end{array} \right)$$ in the same short interval *T*, for obtaining $X_2=f^t (X_1)$ and

$$\begin{aligned} M_2=\left[ u_1^{(2)},\ldots ,u_n^{(2)} \right] =D_{X_1} f^t (M_1 )=\Phi _T (X_1 )\cdot \left[ u_1^{(1)},\ldots ,u_n^{(1)}\right] . \end{aligned}$$

The procedure of integration and orthonormal method repeats for K times.

Through the $k-th$ step, the volume $Vol^p$ changes by a factor of $|| w_1^{(k)}||\ldots || w_p^{(k)}||$, where $\{w_1^{(k)},\ldots ,w_p^{(k)}\}$ is the set of orthogonal vectors calculated from $M_k$ by Gramm-Schmidt method. From (8), we have

14 $$\begin{aligned} \lambda ^p (X_ 0 ,M_ 0 )=\lim _{k\rightarrow \infty }\frac{1}{kT}\sum _{j=1}^k ln \left( || w_1^{(j)}|| \ldots || w_p^{(j)}||\right) . \end{aligned}$$

By subtracting $\lambda ^{p-1}$ from $\lambda ^p$ and (9), we obtain the $p-th$ LCE of order one:

$$\begin{aligned} \lambda _p=\lim _{k\rightarrow \infty }\frac{1}{kT} \sum _{j=1}^{k } ln|| w_p^{(j)}||. \end{aligned}$$

Therefore, for a suitable value of *T*, and a large enough number of iterations *K*, we have the LCE spectra (Sandri 1996),

15 $$\begin{aligned} \lambda _i \approx \frac{1}{KT}\sum _{j=1}^{K} In || w_i^{(j)}||, \quad i=1,2,\ldots ,n. \end{aligned}$$

**Fig. 7:**
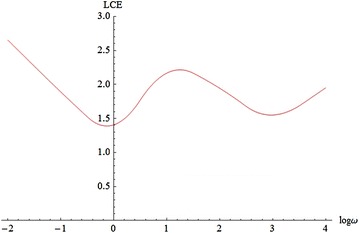
the largest LCEs

The relationship between the largest LCE and parameter $\omega$ of system (4) is shown in Fig. 7, where the horizontal component in $log_{10}w$ and the vertical component is the largest LCEs.

## Synchronization with bidirectional coupling configurations

###  Identical synchronization

In this section, we want to consider the identical synchronization for the chaotic system (4). The possibility of synchronization in a coupled chaotic system composed of identical chaotic oscillators was first reported by Fujisaka and Yamada (1983a, b) and later by Pecora and Carroll (1990). This type of synchronization, identical synchronization (IS), is also known as complete synchronization (CS).

When there are initial conditions so that the systems eventually evolve identically in time, the systems are to be completely synchronized. In bidirectional coupling, both systems are coupled to each other and the coupling factor drives a regulation of the dynamics onto a common synchronized behavior. A linear bidirectional coupling between identical chaotic systems can be discussed as the preliminary of an additional dissipative term in the dynamics of system (10):

16 $$\begin{aligned} \frac{dX}{dt}=F(X)+C.(Y-X) \end{aligned}$$

17 $$\begin{aligned} \frac{dY}{dt}=F(Y)+C.(X-Y) \end{aligned}$$

where *C* is a constant symmetric matrix which describes the strength of the coupling between the oscillators and also is called the interaction matrix. The type of coupling defined by (16) and (17) is also called diffusive coupling (Chen et al. 2011; Kim and Chwa 2011; Pikovsky et al. 2001; Shao et al. 2002).

When one increases the coefficients in systems (16) and (17), a transition to a IS state occurs at a critical value of the coupling. Here, our purpose of the IS state is to be established the asymptotic condition $\lim _{t\rightarrow \infty } || X - Y ||= 0$. In general, the motion of the coupled system, occurs in a phase space of dimension 2*n*. However, when the IS state is achieved, the motion collapses to a subspace $X=Y$ (the synchronization manifold) of phase space. The phase space is combined of two geometrical entities: the synchronization manifold, and the transverse subspace that they are perpendicular subspaces together (Carroll et al. 1997). Here, the systems synchronize in a complete way for all $c>C_T$, that $C_T\cong \frac{\lambda _1}{2}$ is a critical coupling strength and $\lambda _1$ is the largest LCEs.

For the system (4), by using (16), (17), (13), the bidirectional coupled system with diagonal matrix $C = c I$ and $t_1,t_2$ as time variables in Eq. (13), is:

$$\begin{aligned} \dot{x}_1&=\left( 25 cos^2(\omega t_1 ) +10\right) (y_1-x_1 )+c(x_2-x_1);\\ \dot{y}_1 &=\left( 28-35 cos^2(\omega t_ 1 ) \right) x_1-x_1 z_1+ \left( 29 cos^2(\omega t _1 )-1\right) y_1+c(y_2-y_1);\\ \dot{z}_1&=x_1 y_1-\frac{ cos^2(\omega t _1 )+8}{3} z_1+c(z_2-z_1);\\ \dot{t}_1&=1+c(t_2-t_1 ); \\ \dot{x}_2 &=\left( 25 cos^2(\omega {t _2})+10\right) (y_2-x_2 )+c(x_1-x_2); \\ \dot{y}_2 &=\left( 28-35 cos^2(\omega t _2)\right) x_2-x_2 z_2+(29 cos^2(\omega t_2 ) -1) y_2+c(y_1-y_2); \\ \dot{z}_2&=x_2 y_2-\frac{ cos^2(\omega t _2 )+8}{3} z_2+c(z_1-z_2);\\ \dot{t}_2 &=1+c(t_1-t_2 ). \end{aligned}$$

### Synchronized motion and its stability

Now, the important problem here is the stability of the synchronization manifold, the question of what happens when an infinitesimal perturbation occurs in the synchronization manifold. If the perturbation dies off exponentially and the trajectory returns to the synchronization manifold, the synchronized state is said to be stable and it is unstable when the perturbation grows exponentially (Fujisaka and Yamada 1983a). The condition of stability is that the LCEs obtained from the variational equation of the transverse part of the perturbation have to be negative. These are called transverse LCEs that depend on the numerical values of the components of the matrix *C*.

It is evident that the negativity of transverse LCEs represents a necessary condition for the local stability of the synchronized motion. If these are positive, we will never observe the system in its synchronous motion, because perturbations in the vicinity of the manifold would grow exponentially and they have the effect to destroy synchronization (Pecora and Carroll 1990; Carroll et al. 1997).

**Fig. 8:**
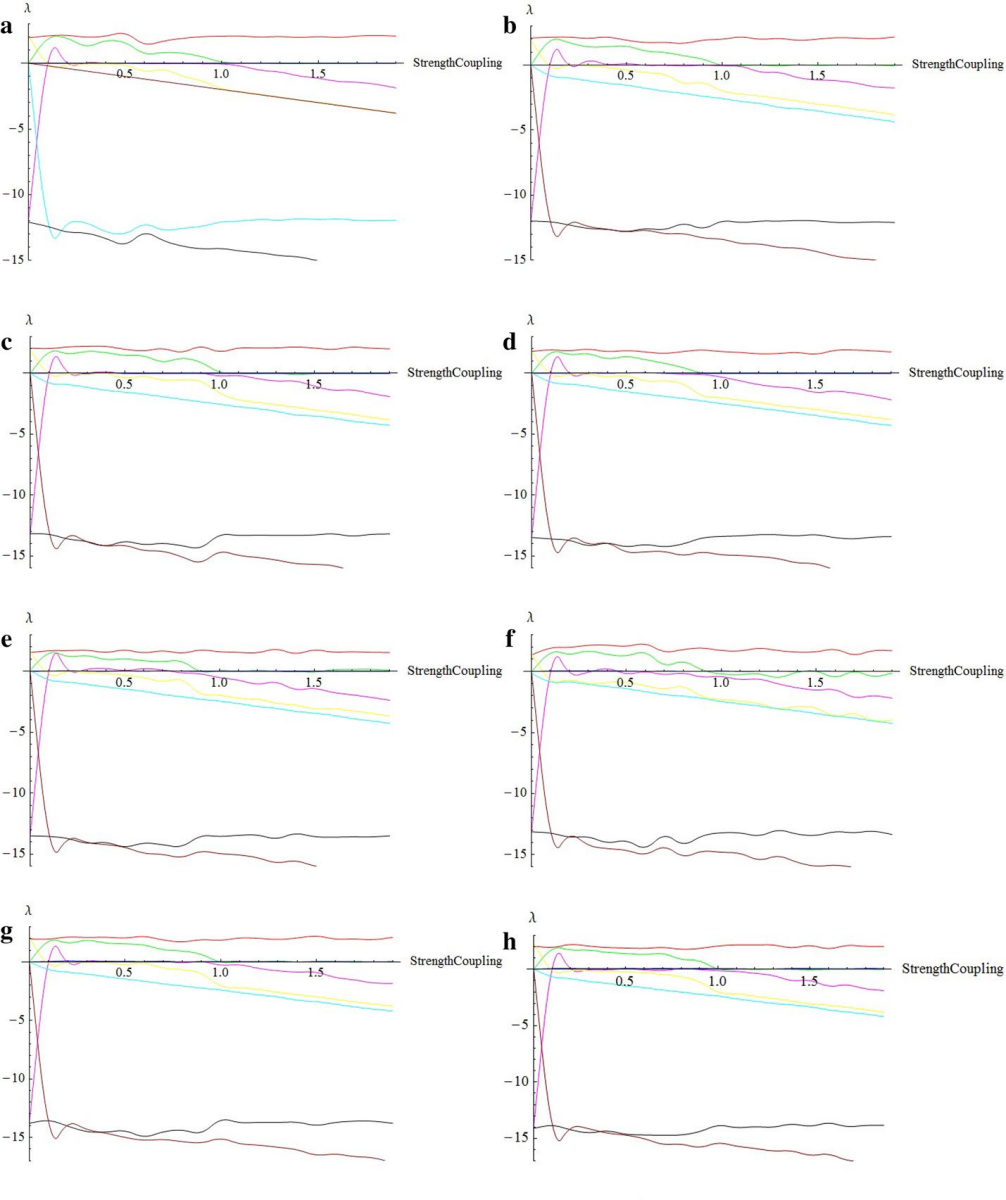
**a** $$\omega =0$$, $$C_T\simeq 1.1$$. **b** $$\omega =0.001$$, $$C_T\simeq 0.98$$. **c** $$\omega =0.01$$, $$C_T\simeq 1.1$$. **d** $$\omega =0.1$$, $$C_T\simeq 0.9$$. **e** $$\omega =1$$, $$C_T\simeq 0.9$$. **f** $$\omega =10$$, $$C_T\simeq 0.92$$. **g** $$\omega =100$$, $$C_T\simeq 0.98$$. **h** $$\omega =1000$$, $$C_T\simeq 1$$

Inspired by the described algorithm in “Estimation of the entire LCE spectrum” section, and by using the program implemented with Mathematica , we calculate the LCE spectra for some of values $\omega$ that illustrated in Fig. 8.

**Fig. 9:**
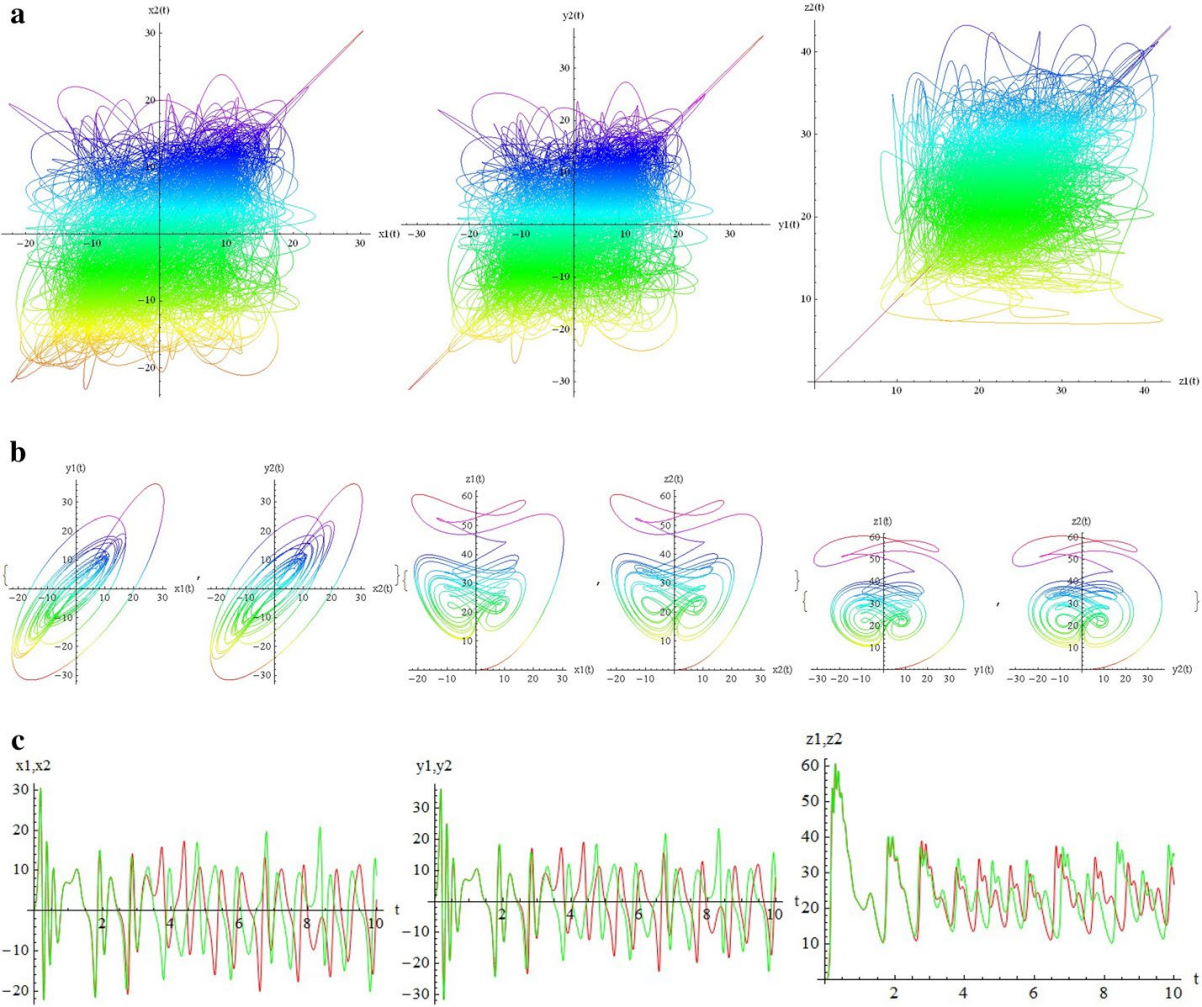
$c= 0 .5<C_T, \quad \omega = 0$. **a** Parametric plots: $x2(x1),\quad y2(y1),\quad z2(z1)$. **b** Parametric plots:$y1(x1),y2(x2),\quad z1(x1),z2(x2),\quad z1(y1),z2(y2),t=10 s$. **c** Time series:$x2(t),x1(t),\quad y2(t),y1(t),\quad z2(t),z1(t),$

**Fig. 10:**
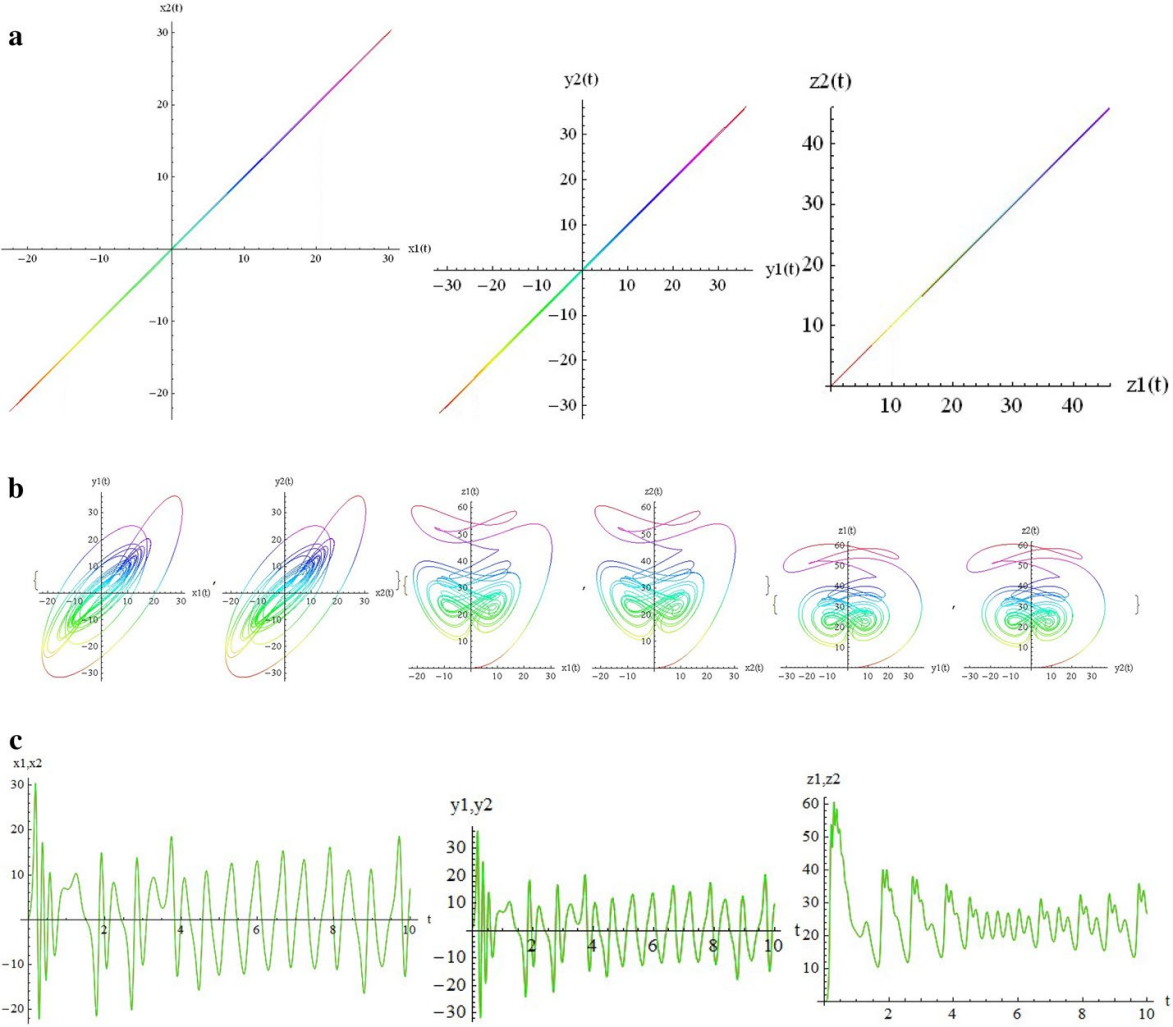
The transition to synchronization $c=1.8>C_T,\quad \omega = 0$. **a** Parametric plots: $x2(x1),\quad y2(y1),\quad z2(z1),$. **b** Parametric plots: $y1(x1),y2(x2), \quad z1(x1),z2(x2),\quad z1(y1),z2(y2),t=10s$. **c** Time series:$x2(t),x1(t),\quad y2(t),y1(t),\quad z2(t),z1(t)$

**Fig. 11:**
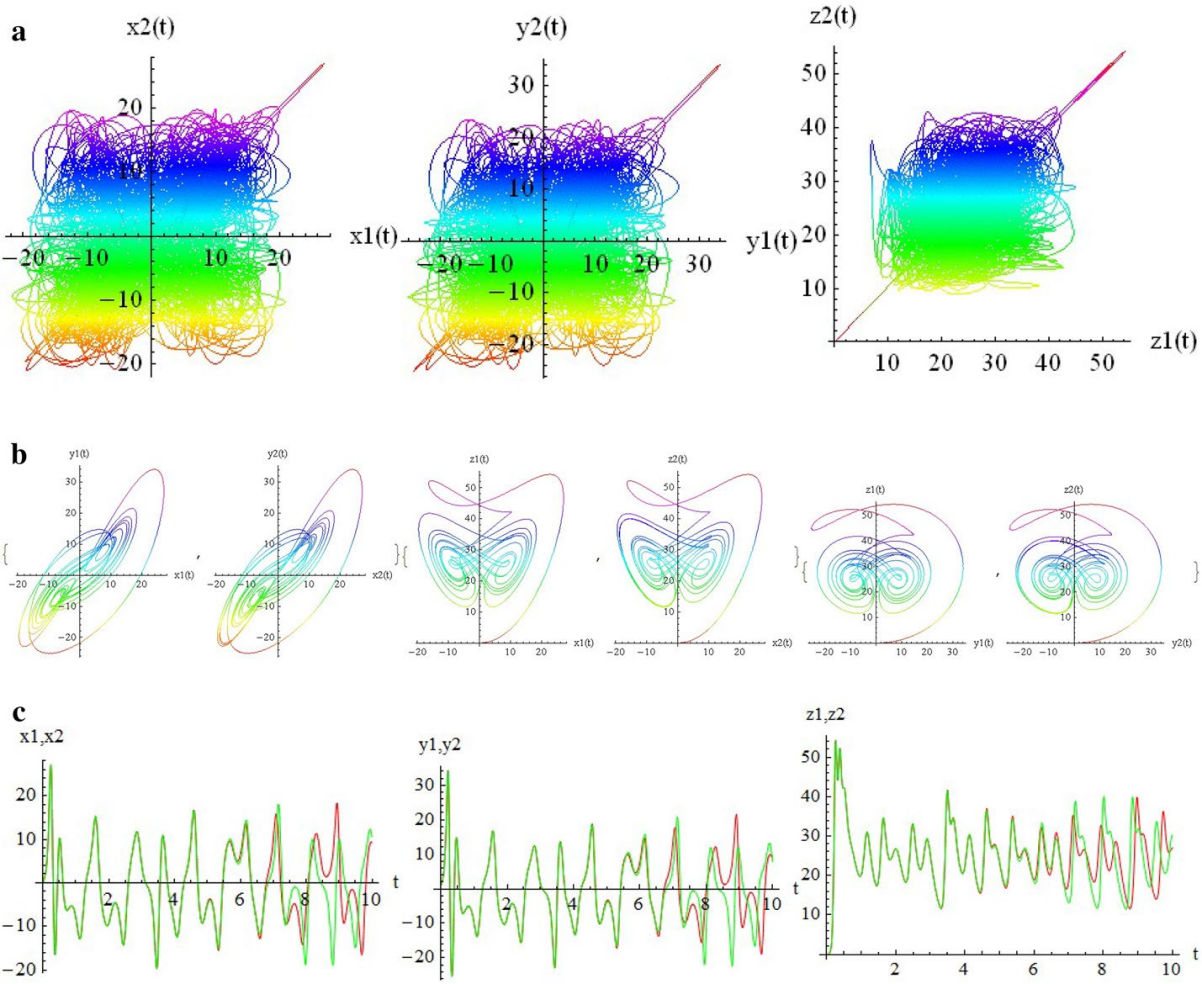
$c= 0 .5<C_T,\quad \omega =1000$. **a** Parametric plots: $x2(x1),\quad y2(y1),\quad z2(z1)$. **b** Parametric plots: $y1(x1),y2(x2), \quad z1(x1),z2(x2), \quad z1(y1),z2(y2),t=10s$. **c** Time series: $x2(t),x1(t),\quad y2(t),y1(t),\quad z2(t),z1(t)$

**Fig. 12:**
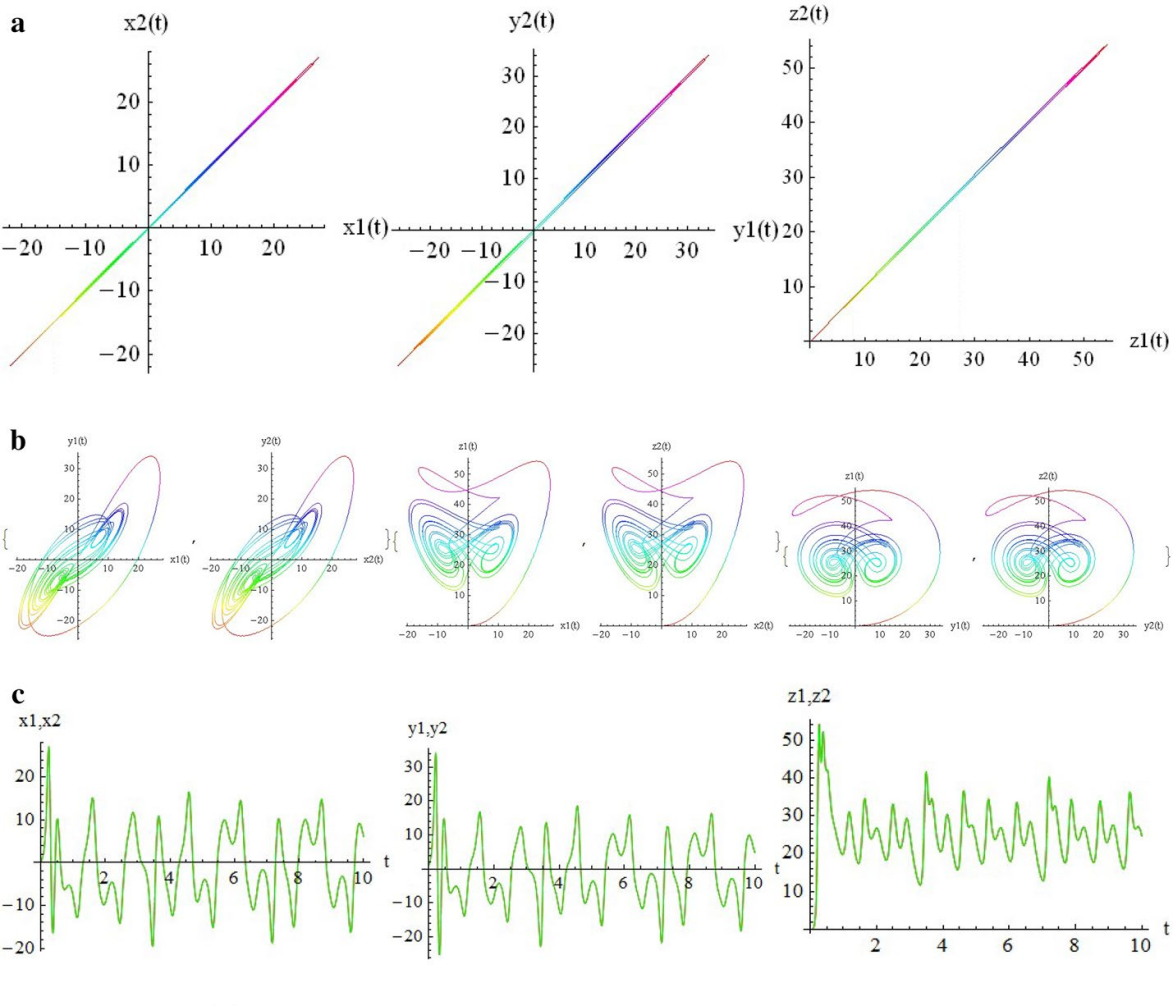
The transition to synchronization $c=1.5>C_T, \quad \omega =1000$. **a** Parametric plots: $x2(x1),\quad y2(y1),\quad z2(z1),$. **b** Parametric plots: $y1(x1),y2(x2),\quad z1(x1),z2(x2),\quad z1(y1),z2(y2),t=10s$. **c** Time series: $x2(t),x1(t), \quad y2(t),y1(t),\quad z2(t),z1(t),$

The transverse LCEs decrease monotonically from the values of LCEs of a single free oscillator by increasing coupling strength. The positive value of LCEs becomes negative at transition value (critical coupling strength), $C_T$. The first value stays positive constant in the whole range of values of *c*. This means that the coupled system stays chaotic even in the asymptotically stable synchronized state. Indeed, it is hyperchaotic below $C_T$, and the transition from hyperchaos to chaos at $C_T$, is the transition to stable synchronization. The motion is restricted to the synchronization manifold for $c>C_T$. The phenomenon of identical synchronization between bidirectionally coupled chaotic systems is illustrated in Figs. 9, 10, 11 and 12.

The simplest way to see the relation between two coupled systems is to plot the variables of one versus (vs.) the variables to the other.The difference between two chaotic states can be also seen from the time series. In Fig. 9, for $\omega = 0$ and below the transition to synchronization (c = 0.5), part (a) demonstrates the parametric plots $x_1$ vs. $x_2$ ($x_2(x_1)$), $y_1$ vs. $y_2$ and $z_1$ vs. $z_2$. In part (b) the components $x_1, y_1, z_1$ of the first system and the components $x_2, y_2, z_2$ of the second system are individually seen pairwise versus each other; for example $x_1$ vs. $y_1$, $x_2$ vs. $y_2$ and so on, for $t = 10 s$. It is not difficult to see the difference between two systems. Part (c) demostrates the two time series $x_1(t)$ (red curve) and $x_2(t)$ (green curve); $y_1(t)$ (red curve) and $y_2(t)$ (green curve); $z_1(t)$ (red curve) and $z_2(t)$ (green curve). In this figures we also demonstrate the sensitivity to small perturbation. Oscillations of components for every two time series have started at different but very close initial conditions.

In Fig. 10, for $\omega = 0$ and above the transition to synchronization (c = 1.8), part (a) demonstrates the parametric plots from the identical synchronization attractor. The states of two systems are identical, as can be easily seen on the planes $x_1$ vs. $x_2$, $y_1$ vs. $y_2$ and $z_1$ vs. $z_2$. The trajectories lie on the diagonal $x_1 = x_2, y_1 = y_2, z_1 = z_2$respectively. Part (b) demonstrates that strong coupling makes the trajectories of two systems nearly identical; for example $x_1$ vs. $y_1$, $x_2$ vs. $y_2$ and so on. Part (c) demonstrates the time series of two systems are chaotic in time, but completely coinciding (red and green curves). Similarly, in Figs. 11 and 12 for w=1000 one can see the status of two coupled systems before and after identical synchronization.

**Fig. 13:**
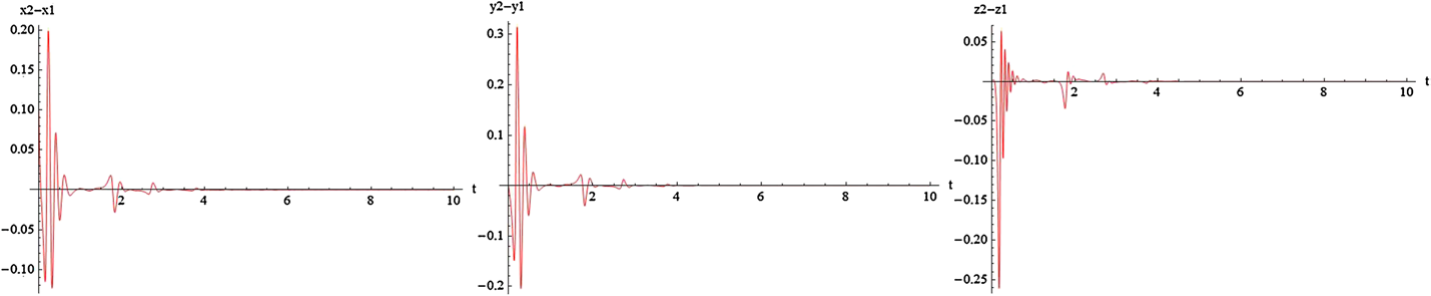
Identical synchronization error $c=1.8>C_T, \quad \omega =0$

**Fig. 14:**
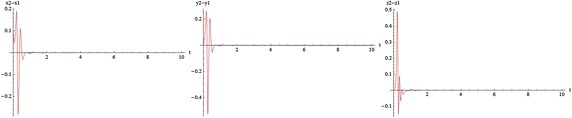
Identical synchronization error $c=1.5>C_T, \quad \omega =1000$

Figure 13, for $\omega = 0$ and $c = 1.8$ and Fig. 14, for $\omega = 1000$ and $c = 1.5$, above the transition to synchronization demonstrate the identical synchronization errors.

## Conclusion

Although there are many well-known autonomous chaotic systems, but a few of non-autonomous have been presented in the articles. This article focuses on another non-autonomous unified chaotic system that is obtained by replacing a fixed parameter in unified chaotic system with a function of time. The system has very rich chaotic dynamical behaviors for varying its parameter. We have studied the special properties of the system in detail. By simulation that performed with mathematica, we have demonstrated that these type of chaotic systems can be synchronized. Then by estimating the LCE spectrum, the synchronized motion and its stability have been studied. The main results of our work in relation to the estimates of the LCE spectrum and the status of the bidirectional coupled systems before and after identical synchronization, for different values of parameters, are shown in two and three dimentional figures beautifully. We think that by replacing the fixed parameter in unified chaotic system with other functions such as unit step function and error function, one can introduce the systems that will be widely applicable in engineering. We will investigate the generalized synchronization between two different systems of this type as soon as possible.
